# Role of *Clostridium perfringens* Enterotoxin on YAP Activation in Colonic Sessile Serrated Adenoma/Polyps with Dysplasia

**DOI:** 10.3390/ijms21113840

**Published:** 2020-05-28

**Authors:** Rina Fujiwara-Tani, Kiyomu Fujii, Shiori Mori, Shingo Kishi, Takamitsu Sasaki, Hitoshi Ohmori, Chie Nakashima, Isao Kawahara, Yukiko Nishiguchi, Takuya Mori, Masayuki Sho, Masuo Kondoh, Yi Luo, Hiroki Kuniyasu

**Affiliations:** 1 Department of Molecular Pathology, Nara Medical University, 840 Shijo-cho, Kashihara, Nara 634-8521, Japan; rina_fuji@naramed-u.ac.jp (R.F.-T.); toto1999-dreamtheater2006-sms@nifty.com (K.F.); shi.m.0310@i.softbank.jp (S.M.); nmu6429@yahoo.co.jp (S.K.); takamitu@fc4.so-net.ne.jp (T.S.); brahmus73@hotmail.com (H.O.); c-nakashima@naramed-u.ac.jp (C.N.); isao_kawahara@a011.broada.jp (I.K.); yukko10219102@yahoo.co.jp (Y.N.); pt_mori_t@yahoo.co.jp (T.M.); 2 Department of Surgery, Nara Medical University, 840 Shijo-cho, Kashihara, Nara 634-8522, Japan; m-sho@naramed-u.ac.jp; 3 Drug Innovation Center, Graduate School of Pharmaceutical Sciences, Osaka University, 6-1 Yamadaoka, Suita, Osaka 565-0871, Japan; claudindds@gmail.com; 4 Key Laboratory of Neuroregeneration of Jiangsu and Ministry of Education, Co-Innovation Center of Neuroregeneration, Nantong University, Nantong 226001, Jiangsu Province, China

**Keywords:** clostridium perfringens enterotoxin, claudin-4, YAP, Hippo signal, SSA/P

## Abstract

Sessile serrated adenoma/polyp with dysplasia (SSA/P-D) is an SSA/P with cellular dysplasia and has a higher risk of progressing to colon carcinogenesis. Previously, we reported that tight junction impairment by *Clostridium* *perfringens* enterotoxin (CPE) leads to activation of the transcriptional co-activator yes-associated protein (YAP) in oral squamous cell carcinoma. Here, we investigated whether CPE activates YAP to promote the malignant progression of SSA/P. E-cadherin expression was lower in the 12 cases with SSA/P-D examined than that in normal mucosa, SSA/P, or tubular adenoma (TA). Furthermore, intracellular translocation of claudin-4 (CLDN4) and nuclear translocation of YAP were observed. The CPE gene was detected in DNA extracted from SSA/P-D lesions, but not in SSA/P or TA. Treatment of the rat intestinal epithelial cell line IEC6 with low-dose CPE resulted in intracellular translocation of CLDN4 to the cytoplasmic membrane. Cytoplasmic CLDN4 showed co-precipitation with transcriptional co-activator with PDZ-binding motif, zonula occludens (ZO)-1, large tumor suppressor, and mammalian Ste20-like. Additionally, YAP co-precipitated with ZO-2 under CPE treatment led to decreased YAP phosphorylation and nuclear translocation. YAP activation promoted increase in nuclear TEA domain family member level, expression of cyclin D1, snail, vimentin, CD44, NS and decrease in E-cadherin levels, thereby inducing stemness and epithelial-mesenchymal-transition (EMT). The Hippo complex with the incorporation of CLDN4 increased stability. Upon low-dose CPE treatment, HT29 cells with BRAF^V600E^ gene mutation showed increased growth, enhanced invasive potential, stemness, and induced EMT phenotype, whereas HCT116 cells, which carry KRAS^G13D^ gene mutation, did not show such changes. In an examination of 10 colorectal cancers, an increase in EMT and stemness was observed in CPE (+) and BRAF mutation (+) cases. These findings suggest that *C.* *perfringens* might enhance the malignant transformation of SSA/P-D via YAP activation. Our findings further highlight the importance of controlling intestinal flora using probiotics or antibiotics.

## 1. Introduction

Sessile serrated adenoma/polyp (SSA/P) has attracted attention as a frequently occurring precancerous lesion during cancer development and is considered to progress to cancer via SSA/P with dysplasia (SSA/P-D) [1,2,3]. SSA/P-D is noted as a direct precancerous lesion of colorectal cancer (CRC). SSA/P-D is clinically older, larger in size, and has been reported to have endoscopic characteristics of semipedunculated morphology, double elevation, central depression, reddishness, and adenomatous pit pattern [4,5]. 

RAS mutations, which are commonly found in tubular and tubulovillous adenoma, are rare genetic abnormalities in SSA/P, whereas CpG island methylator phenotype positive (MLH1 negative), microsatellite instability status, and BRAF mutations are known as common gene profiles of SSA/P [6], which is emphasized in the progression to CRC along with DNA mismatch repair gene abnormalities [7]. In addition, mutations in *FBXW7*, *TP53*, *KIT*, *PTEN*, and *SMAD4* genes are also reported [8]. In this study we focused on the Hippo pathway as being involved in the progression of SSA/P-D.

The Hippo signaling system is a multifunctional process associated with development, homeostasis, regeneration, and diseases [9]. This pathway is known to be controlled by the cellular status, such as cell-cell adhesion, cytoskeleton, and energy metabolism [10]. In mammals, the Hippo pathway proteins mammalian Ste20-like (MST) and large tumor suppressor (LATS) suppress the transcriptional co-activators yes-associated protein (YAP) and transcriptional co-activator with PDZ-binding motif (TAZ), which interact with TEA domain family member (TEAD) to regulate expression of genes that control proliferation, survival, differentiation, and also cancer development [11]. As cell adhesion activates MST, the deletion of E-cadherin, which forms an adherens junction, activates YAP [12]. Like adherens junction proteins, the tight junction proteins angiomotin and ZO-2 sequester YAP/TAZ and inhibit its activity [13,14]. In CRC, overexpression of the tight junction protein claudin-4 (CLDN4) is observed, which is a target of *Clostridium perfringens* enterotoxin (CPE), a toxin of the intestinal flora [15,16,17]. High concentration of c-terminus of CPE binds to second extracellular loop of CLDN4 to destruct homotipic claudin bindings of the tight junction to provide diarrhea [16]. In contrast, we have reported that impairment of tight junction by low concentration of CPE leads to activation of YAP by internalization of CLDN4 in oral squamous cell carcinoma (OSCC) [18]. In the present study, we investigated whether such CPE-mediated activation of YAP is involved in the malignant progression of SSA/P and malignant phenotype of CRC, which findings might be expected to provide prevention of CRC. 

## 2. Results

### 2.1. Increase in Cytoplasmic CLDN4 Level in SSA/P-D

**Figure 1:**
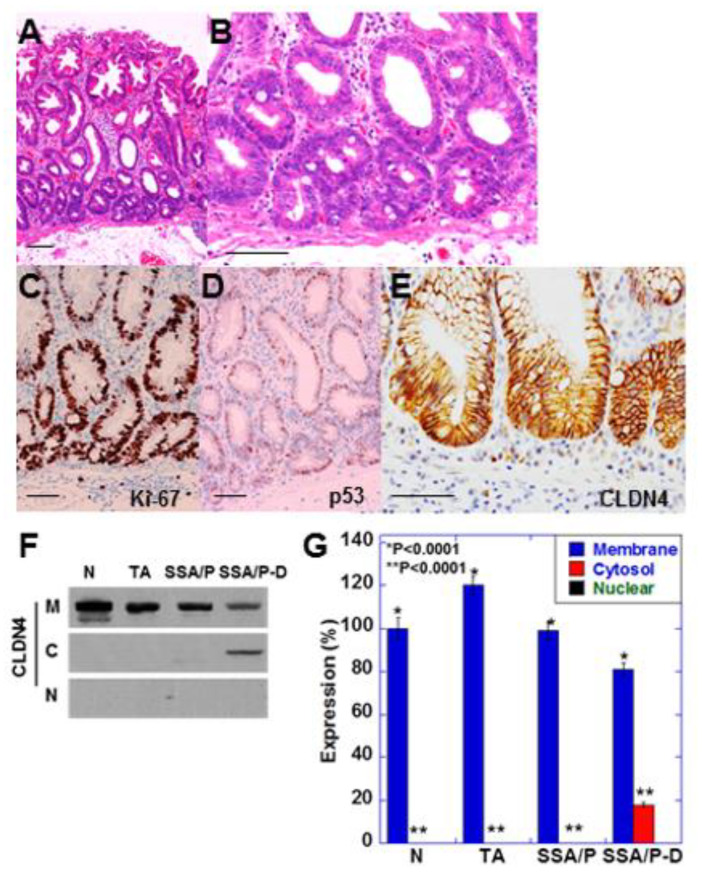
Expression of CLDN4 in SSA/P-D.(**A**,**B**) Histopathological feature of SSA/P-D, (**C**–**E**) Protein expression of Ki-67 (**C**), p53 (**B**) and CLDN4 in the SSA/P-D lesion. Scale bar, 100 μm. (**F**,**G**) Subcellular localization of CLDN4 in SSA/P-D by western blot analysis. N, normal mucosa; TA, tubular adenoma; SSA/P, serrated sessile adenoma/polyp; SSA/P-D, SSA/P with dysplasia; M, membrane fraction; C, cytosolic fraction; N, nuclear fraction; Error bar, standard deviation from 3 independent trials.

SSA/P-D showed cytological atypia in bottom of the serrated glands and pseudostratification of swollen nuclei (Figure 1A,B). These atypical cells showed increased Ki-67 positivity and weak accumulation of p53 (Figure 1C,D). Furthermore, immunostaining revealed weak localization of CLDN4 in the cytoplasm as well as in the cell membrane (Figure 1E). Cytoplastic CLDN4 was confirmed by immunoblotting (Figure 1F,G). In contrast, cytoplasmic CLDN4 was not detected in the normal mucosa, tubular adenomas, and SSA/P samples.

### 2.2. Epithelial-Mesenchymal-Transition (EMT) Phenotype and YAP Activation in SSA/P-D

**Figure 2:**
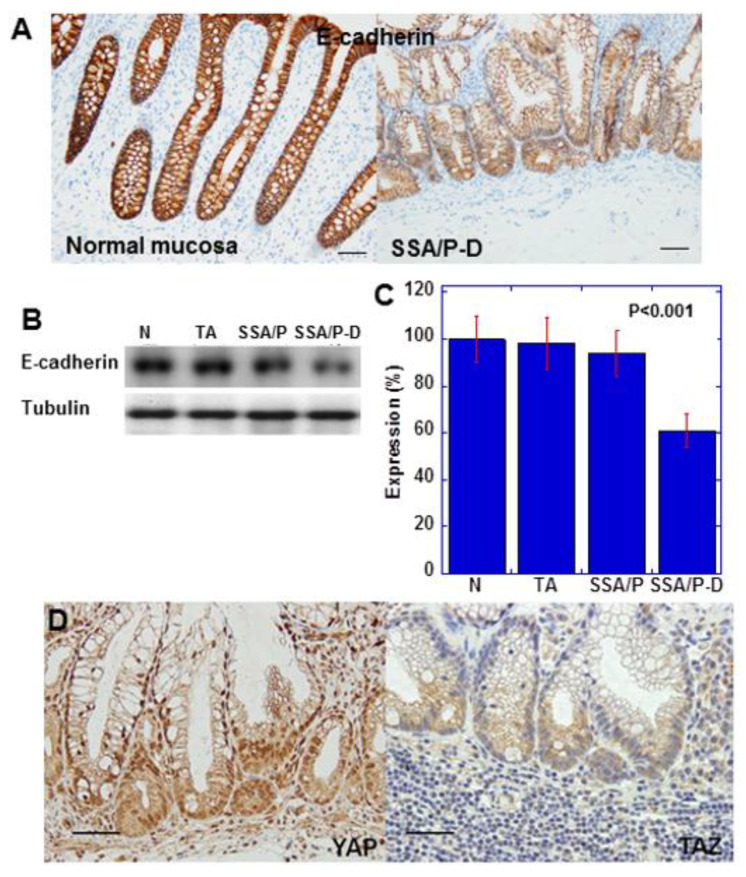
Expression of E-cadherin and YAP in SSA/P-D. (**A**) E-cadherin protein expression compared between normal mucosa and SSA/P-D. (**B**,**C**) E-cadherin protein levels detected by western blot analysis. (**D**) YAP and TAZ protein expression examined by immunohistochemistry. Scale bar, 100 μm; N, normal mucosa; TA, tubular adenoma; SSA/P, serrated sessile adenoma/polyp; SSA/P-D, SSA/P with dysplasia; Error bar, standard deviation from three independent trials.

E-cadherin expression in SSA/P-D was reduced compared with that in normal mucosa (Figure 2A). Moreover, immunoblotting indicated reduced E-cadherin protein level in SSA/P-D compared with that in normal mucosa, tubular adenomas, and SSA/P (Figure 2B,C). In the atypical cells of SSA/P-D, nuclear localization of YAP, but not of TAZ, was observed (Figure 2D). Notably, 10 out of 12 cases with SSA/P-D showed decreased E-cadherin and 9 cases showed cytoplasmic CLDN4 and nuclear YAP1, but not nuclear TAZ. In contrast, decreases in E-cadherin, cytoplasmic CLDN4, and nuclear YAP1 were not found in normal mucosa, tubular adenomas, and SSA/Ps (Table 1).

### 2.3. Effect of CPE on Colon Epithelial Cells

**Figure 3:**
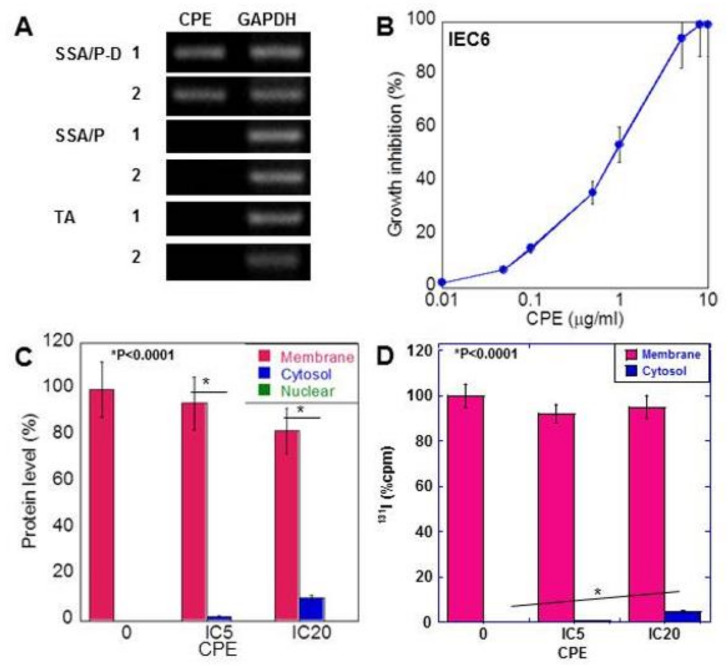
Effect of CPE on IEC6 rat intestinal epithelial cells. (**A**) CPE gene was amplified by PCR using DNA extracted from paraffin-embedded specimens. (**B**) Effect of CPE on growth of IEC6 cells. (**C**) Subcellular localization of CLDN4, examined by ELISA. (**D**) Subcellular localization of CLDN4, which was labeled by conjugation of ^131^I onto cell surface protein. CLDN4 protein was examined by liquid scintillation counter. Error bar, standard deviation from 3 independent trials. CPE, *Clostridium perfringens* enterotoxin; TA, tubular adenoma; SSA/P, serrated sessile adenoma/polyp; SSA/P-D, SSA/P with dysplasia; M, membrane fraction; C, cytosolic fraction; N, nuclear fraction; IC, inhibitory concentration.

Non-membranous CLDN4 localization is reported to be mediated by CPE [18]. Therefore, we examined *CPE* gene expression in the adenoma tissues (Figure 3A). *CPE* gene was induced in the SSA/P-D samples but was not detected in tissues of SSA/P and tubular adenoma. As shown in Table 2, *CPE* gene was amplified by PCR in 11 out of 12 SSA/P-D cases, in only 1 out of 25 SSA/P cases, and in none of the normal mucosa and tubular adenomas.

In rat colon epithelial cells, CPE showed growth inhibition in a dose-dependent manner (Figure 3B). Treatment with low-dose CPE (IC 5 and IC 20) resulted in a decrease in membranous CLDN4 and increase in cytoplasmic CLDN4 in a dose-dependent manner (Figure 3C). In contrast, nuclear localization of CLDN4 was not detected.

To confirm translocalization of membranous CLDN4 to cytoplasm, cell surface protein labeling was carried out using ^131^I and labeled CLDN4 was examined in low-dose CPE-treated IEC6 cells (Figure 3D). The ^131^I-labeled CLDN4 was detected in the cytoplasm of these cells in a dose-dependent manner.

### 2.4. Protein-Protein Interaction of Cytoplasmic CLDN4

**Figure 4:**
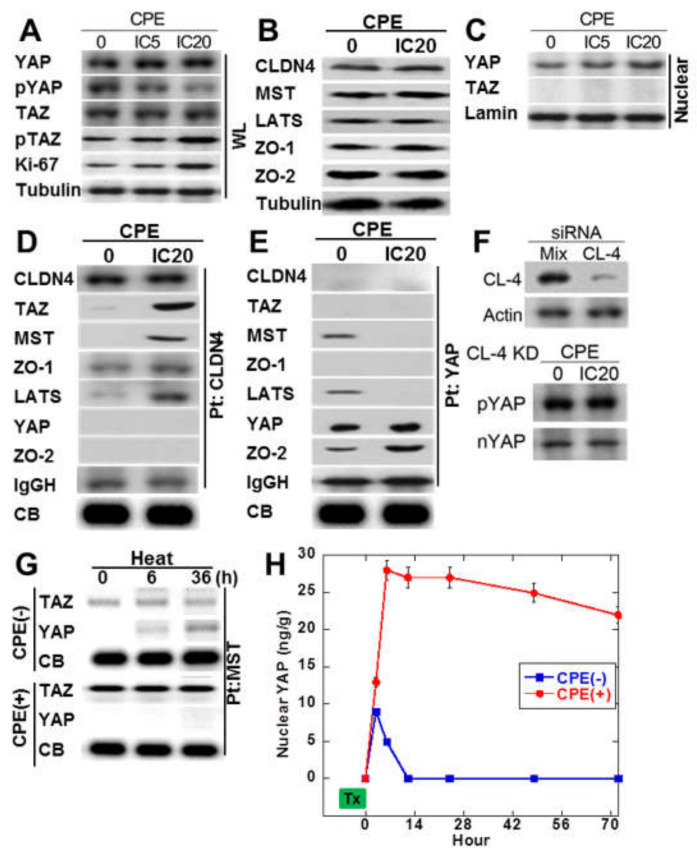
Effect of CPE on Hippo/YAP signal in IEC6 cells. (**A**) Effect of CPE on YAP phosphorylation. (**B**) Effect of CPE on protein levels of CLDN4, Hippo proteins (MST, LATS), and CLDN4-associated proteins (ZO-1 and ZO-2). (**C**) Effect of CPE on nuclear translocation of YAP. (**D**) Effect of CPE on protein association with CLDN4. Immunoprecipitation by anti-CLDN4 antibody was detected using antibodies against CLDN4 and Hippo proteins. (**E**) Effect of CPE on protein association with YAP. Immunoprecipitation by anti-YAP antibody was detected using antibodies against CLDN4 and Hippo proteins. (**F**) Effect of CLDN4 knockdown on phosphorylation and nuclear translocation of YAP. (**G**) IEC cells pretreated with 42 °C or 42 °C + CPE (IC 20) for 30 min were examined MST-bound TAZ or YAP temporally by immunoprecipitation. (**H**) Nuclear YAP was examined by ELISA in IEC6 cells pretreated with 42 °C or 42 °C + CPE. CPE, *Clostridium perfringens* enterotoxin; Pt, immunoprecipitation; IC, inhibitory concentration; IgGH, immunoglobulin G heavy chain; CB, coomassie blue staining of a slot blot of the protein used for immunoprecipitation; WL, whole cell lysate; Nuclear, nuclear fraction; nYAP, nuclear YAP; KD, knockdown. Error bar, standard deviation from 3 independent trials.

Since immunostaining of SSA/P-D showed nuclear YAP immunoreactivity, phosphorylation levels of YAP and TAZ in CPE-treated IEC6 cells (Figure 4A). Total protein levels of YAP and TAZ were not affected by CPE treatment. Phosphorylated YAP was decreased, whereas phosphorylated TAZ was not altered. As shown in Figure 4B, MST and LATS in Hippo suppression system were not altered by CPE treatment. ZO-1 and ZO-2 of tight junction-lining proteins were also not altered by CPE treatment. As shown in Figure 4C, decrease in phosphorylated YAP and increase in nuclear YAP were observed in CPE-treated IEC6 cells. To examine the protein-protein interaction of cytosolic CLDN4, immunoprecipitation was performed in CPE-treated IEC6 cells in Figure 4D. Immunoprecipitant of anti-CLDN4 antibody showed co-precipitation with TAZ, MST, ZO-1, and LATS, but not with YAP or ZO-2 in CPE-treated IEC6 cells. In contrast, the untreated IEC6 cells did not show co-precipitation of CLDN4 with TAZ, MST, and LATS. In CPE-treated IEC6 cells, immunoprecipitant of anti-YAP antibody showed co-precipitation with ZO-2, but not with CLDN4, TAZ, MST, ZO-1, LATS (Figure 4E). In contrast, the untreated IEC6 cells showed co-precipitation of YAP with MST and LATS. When CLDN4 was knocked down, nuclear translocation or phosphorylation of YAP were not altered (Figure 4F). 

We next examined the effect of CPE on the Hippo system via CLDN4 in a view of temporality (Figure 4G,H). Examining the time course of TAZ or YAP, which forms a complex with MST by treating cells with heat alone or heat + CPE, the heat treatment alone temporarily decreases the YAP-MST complex and activates YAP. On the other hand, in treatment with heat + CPE, MST forms a strong complex with TAZ, and no complex formation with YAP is observed. 

### 2.5. Effect of Low-Dose CPE on IEC6 Cells

**Figure 5:**
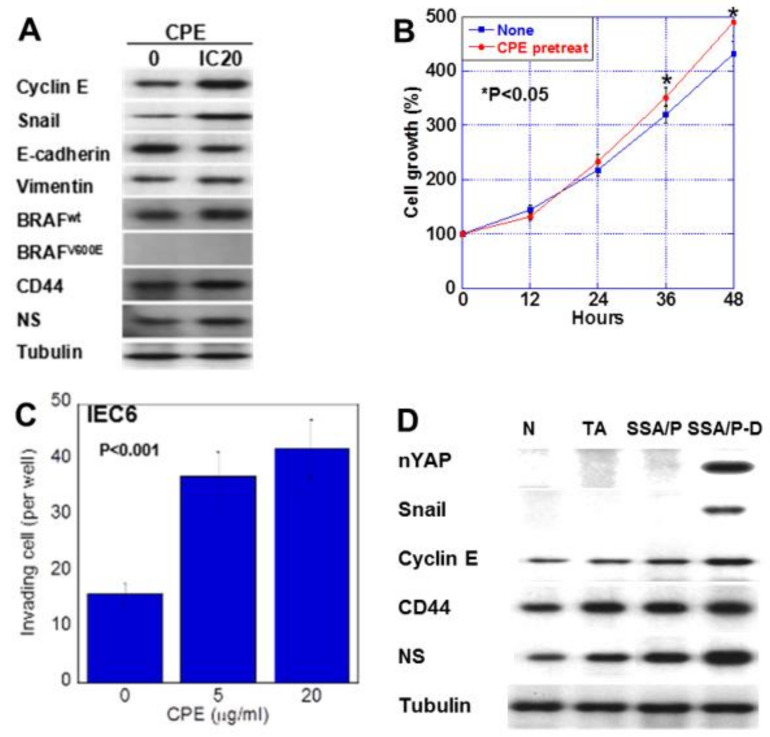
Effect of CPE on EMT phenotype in IEC6 cells and SSA/P-D. (**A**) Effect of CPE on protein levels of factors associated with EMT, stemness, and BRAF. (**B**) Effect of CPE pretreatment on cell growth. IEC6 cells were treated with CPE of IC5 for 24 h. (**C**) Effect of CPE on cell invasion. (**D**) Protein levels of nuclear YAP (nYAP), and factors associated with EMT or stemness were compared in normal mucosa (N), tubular adenoma (TA), SSA/P and SSA/P-D. Error bar, standard deviation from 3 independent trials. CPE, clostridium perfringens enterotoxin; EMT, epithelial-mesenchymal-transition.

We next examined the effect of low-dose CPE on proliferation and invasion-related protein expression in IEC6 cells (Figure 5). CPE treatment increased the expression of cyclin E, snail, vimentin, CD44 and NS; however, it decreased E-cadherin levels (Figure 5A). Since SSA/P-derived dysplasia and adenocarcinoma, BRAF mutation is reported to show high incidence [19], we examined effect of CPE on BREF expression. CPE treatment increased the wild type BRAF expression in IEC6 cells. Notably, although CPE treatment suppressed cell growth, it increased cell invasion (Figure 5B,C). In the CPE-treated cells, cell growth suppression due to CPE toxicity seems to exceed YAP activation. However, the growth rate after CPE treatment is higher than the untreated group. This is thought to be due to YAP activation. To assess the effect of CPE, nuclear YAP levels and the expression of cyclin E, snail, CD44 and NS were examined in in normal mucosa, tubular adenomas, SSA/P and SSA/P-D (Figure 5D). Nuclear YAP and snail were found in only SSA/P-D. Cyclin E, CD44 and NS were increased in SSA/P-D.

### 2.6. Effect of Low-Dose CPE on Human CRC Cell Lines

**Figure 6:**
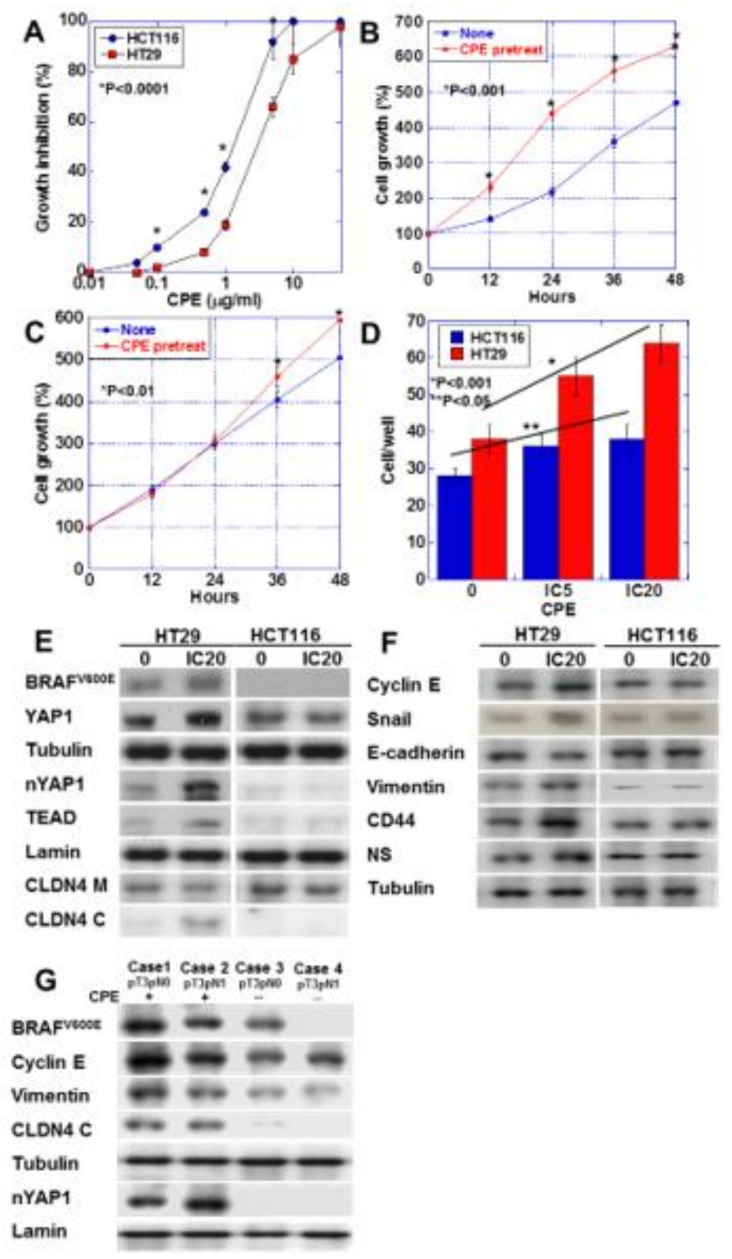
Effect of CPE on EMT phenotype in HT29 and HCM116 human colon cancer cells. (**A**) Effect of CPE on cell growth in the two cell lines. (**B**,**C**) Effect of CPE pretreatment on cell growth. HT29 cells (**B**) and HCT116 cells were treated with CPE of IC5 for 24 h. (**C**). (**D**) Effect of CPE on cell invasion. (**E**) Effect of CPE on protein levels of factors associated with BRAF, Hippo pathway, and CLDN4. (**F**) Effect of CPE on protein levels of factors associated with EMT and stemness. (**G**) BRAF mutation, nuclear YAP and protein levels of factors associated with EMT were examined in 4 CRC cases with or without CPE expression. Error bar, standard deviation from 3 independent trials. CLDN4 M, membranous CLDN4; CLDN4 C, cytosolic CLDN4; EMT, epithelial-mesenchymal-transition; IC, inhibitory concentration.

We nest examined the effect of CPE on HT29 CRC cells carrying BRAF^V600E^ mutation/wild type RAS and HCT116 CRC cells carrying wild type BRAF and KRAS^G13D^ mutation [20,21] (Figure 6). These cell lines lack microsatellite instability; however, we used these cell lines to investigate the association of CPE-CLDN4-YAP in CRCs derived from adenomas, which accounted for the majority. HT29 cells showed lower sensitivity to CPE than HCT116 cells (Figure 6A). Interestingly, low-dose CPE promoted HT29 cell growth, but suppressed HCT116 cell growth (Figure 6B,C). Low-dose CPE enhanced invasive activity in both CRC cells, although HT29 cells showed more pronounced invasion upon CPE treatment than the HCT116 cells (Figure 6D). Activation of YAP, cytosolic CLDN4, and increase in BRAF^V600E^ were detected in CPE-treated HT29 cells. In contrast, CPE-treated HCT116 showed no activation of YAP (Figure 6E). Unlike the HCT116 cells, the CPE-treated HT29 cells showed increased expression of cyclin E, snail, vimentin, CD44, and NS, and decreased expression of E-cadherin (Figure 6F). This suggests that HT29 cells, but not HCT116 cells, show EMT phenotype and increased stemness upon CPE treatment. In order to confirm whether the results of our study in CRC cell lines were also observed in human CRC cases, we examined 10 CRC cases (Figure 6G and Table 3). As a result, CRC cases with BRAF mutation showed CLDN4 cytoplasmic translocation and YAP nuclear translocation when CPE was positive. In contrast, such changes were not observed when the BRAF mutation was negative or CPE was negative. 

## 3. Discussion

In the present study, we observed CPE affected CLDN4 intracellular localization, which is associated with YAP activation. SSA/P is currently a focus of attention as a high-risk lesion of colon carcinogenesis, and SSA/P-D is considered to be a precancerous lesion due to accumulation of increased abnormal phenotypes in SSA/P [1,2,3].

In this study, the SSA/P-D cases had a characteristic *C. perfringens* infection, but not the SSA/P or tubular adenoma cases. *C. perfringens* dysbiosis causes mucosal permeability enhancement, endotoxemia, insulin resistance, systemic inflammation, adiposity, and CRC. Infection of *C. perfringens* is considered as a risk for colon carcinogenesis along with metabolic disorders such as type 2 diabetes mellitus, non-alcoholic steatohepatitis, and irritable bowel disorder [22]. Experimentally, contamination of *C. perfringens* in the intestine of a bacterium-free rat with azoxymethane-induced colon carcinogenesis resulted in a 2.8-fold increase in cancer foci compared with that in control rats [23]. In fact, CRC patients display bacteremia due to several species such as *Fusobacterium nucleatum*, *Peptostreptococcus* species, *Clostridium septicum*, *C. perfringens*, and *Gemella morbillorum* [24]. Additionally, *F. nucleatum* and *Clostridium difficile* in feces are higher in such patients than in healthy subjects. These findings suggest that abnormal intestinal flora, including *C. perfringens*, increase the risk of colon carcinogenesis, as compared to those in the healthy population [25]. Conversely, suppression of intestinal *C. perfringens* and *C. difficile* inhibits colon carcinogenesis in APC-deficient mice [26]. The mechanisms of carcinogenesis promotion by *C. perfringens* have been suggested, including increase in promoter DNA methylation [27] and induction of production of inflammatory cytokines such as tumor necrosis factor-α and interleukin-12 [28]. In this study, we attempted to clarify the mechanism of YAP activation by CPE.

The C-terminus domain of CPE (C-CPE) binds to the first and second extracellular loops of CLDN3 and CLDN4 and destroys the tight junction structure [16,17]. In our study, intracellular CLDN4 translocation was observed following the destruction of tight junctions by CPE. So far, CPE and C-CPE have been reported to cause intracellular translocation of the cytoplasmic membrane CLDN4 in colon and ovarian cancer cells [29,30]. In addition, intracellular CLDN4 translocation has been observed in rat reflux esophagitis model [31] and estrogen treatment of endometrial cancer cells [32,33]. Phosphorylation of CLDN4 by PKC or EphA2 impairs tight junctions and leads to translocation of CLDN4 into the cytoplasm [34,35]. Furthermore, CLDN4 translocated into cytoplasm has been suggested to be involved in trafficking and signaling [36].

Our data indicated that CLDN4 formed a complex with TAZ and the Hippo repression system in the cytoplasm, leading to YAP activation. CLDN4 changes whether YAP or TAZ is incorporated into the Hippo suppression complex. The preferential binding of TAZ by the Hippo complex might provide YAP releasing from the complex, which leads to YAP activation. The relationship between CLDN protein and the Hippo proteins has been reported; CLDN6 activates YAP and induces EMT by binding to LATS and inhibiting YAP suppression in gastric cancer cells [37]. Conversely, in the lung, CLDN18 of epithelial stem cells binds to and inactivates YAP, thereby suppressing lung carcinogenesis [38]. Apart from CLDN, ZO-1 overexpression is also reported to be involved in TAZ/TEAD activation in multiple myeloma, and occludin and YAP/TEAD was reported to bind in pancreatic ductal epithelium and pancreatic cancer [39,40].

In the current study, we showed that YAP formed a complex with ZO-2 and translocated into the nucleus, while CLDN4-ZO-1-MST-LATS interacted with TAZ to form a complex in the cytoplasm. Under normal conditions, ZO-2 promotes the Hippo suppression system [14,41]. In contrast, the YAP-ZO-2 complex promotes YAP nuclear translocation and activates YAP [42,43]. Upon CPE treatment, YAP was found to be activated, but TAZ was inactivated, indicating a difference between the roles of YAP and TAZ. The upstream mechanism that differentially controls YAP and TAZ is unclear [44]. Our data showed that that the addition of CLDN4 to the Hippo repression complex increased the affinity for TAZ and extended the continuity of the complex. TAZ and YAP show different responses to oxidative stress. In TAZ, oxidative stress stabilizes and activates molecules by S-glutathionylation of cystine residues, whereas YAP does not show such a reaction [45]. Hypoxia reduces YAP phosphorylation, whereas TAZ shows increased phosphorylation under such conditions. In other words, YAP tends to be activated in a hypoxic environment [46]. The colonic mucosa is exposed to a hypoxic environment [47]. It has been suggested that YAP might be preferentially activated in a hypoxic environment wherein *C. perfringens* proliferates. In oral cancer, YAP is the target of activation by *C. perfringens*, suggesting the involvement of hypoxia [18].

In our data, CPE DNA was detected characteristically in SSA/P-D tissues. This suggests that *C. perfringens* in SSA/P lesions activated YAP and enhanced atypia. On the other hand, properties of the secreted mucus from the lesion might be altered when SSA/P becomes SSA/P-D, which might exacerbate *C. perfringens* infection. In any case, it is predicted that the malignant phenotype is further enhanced in SSA/P-D by *C. perfringens* growth.

CPE damages epithelial cells [48]. In all of the cells examined in this study, namely IEC6, HT29 and HCT116 cells, CPE showed dose-dependent cytotoxicity. However, CPE enhances growth ability (Ki-67 levels), which suspects increased cell turnover in IEC6 cells. Our results showed low concentration of CPE induced stemness and EMT in IEC6 cells. These are important phenotypes in malignant transformation. Although CPE causes weak growth suppression, it is considered to play an important role in the acquisition of malignant phenotype. In contrast, HT29 cells with BRAF^V600E^ mutation and no microsatellite instability showed lower sensitivity to CPE, and more pronounced invasive ability, stemness and EMT phenotype than the HCT116 cells with KRAS^G13D^ mutation and microsatellite instability. Although both BRAF mutation and microsatellite instability are considered as molecular markers for serrated colorectal lesions, BRAF mutation might provide low sensitivity to CPE cytotoxicity and high responsibility to YAP activation. This association was also found in CRC cases. A link between BRAF^V600E^ mutation and YAP activation has also been found in thyroid cancer [49]. In cancers with the BRAF^V600E^ mutation, activation of YAP promotes resistance to RAF and MEK inhibitors [50]. CPE-induced YAP activation promoted stemness and EMT. Indeed, lymphatic invasion and lymph node metastasis are common in SSA/P-derived cancers [51]. Our data suggest that the synergistic effect of BRAF gene mutation and YAP activation by CPE might promote malignant phenotypes not in SSA/P-D but also in non-serrated type CRCs, especially CRCs possessing BRAF mutation. 

Together, these results suggest that CPE is involved in the opposing effects of cytotoxicity and YAP activation. These effects might be related to malignant transformation of SSA/P-D and BRAF mutation. However, these schemes have not been fully proved in the present study. In future, experiments using appropriate animal models and intestinal organoids will be necessary. This further suggests the importance of controlling intestinal flora by probiotics or antibiotics, and [52], and the need for developing vaccine against *C. perfringens* [53] for the prevention and suppression of progression of SSA/P-D-derived CRC. 

## 4. Materials and Methods

### 4.1. Surgical Specimens

We reviewed the pathological diagnosis and clinical data of all 105 patients diagnosed with non-pathological change (40 cases), tubular adenoma (28 cases), SSA/P (25 cases), and SSA/P-D (12 cases) from 2004 to 2016, in the Department of Molecular Pathology, Nara Medical University, Japan. As written informed consents were not obtained from the patients in this retrospective study, any identifying information was removed from the samples prior to analysis, to ensure strict privacy protection (unlinkable anonymization). All procedures were performed in accordance with the Ethical Guidelines for Human Genome/Gene Research issued by the Japanese Government and were approved by the Ethics Committee of Nara Medical University (approval number 937, 1/4/2011).

### 4.2. Human Cell Lines

HT29 and HCT116 human OSCC cell lines were purchased from Dainihon Pharmaceutical Co. (Tokyo, Japan). IEC rat intestinal epithelial cell line was kindly provided from Professor I.J. Fidler (MD Anderson Cancer Center, Houston, Tx, USA) [54]. Cells were cultured in Dulbecco’s modified Eagle’s medium supplemented with 10% fetal bovine serum at 37 °C in 5% CO_2_. Cell growth was assessed using tetrazolium (MTT) dye assay, as previously described [55].

### 4.3. Antibody and Reagents

The anti-human CLDN4 extracellular domain antibody, 4D3, was developed by immunizing rats with a plasmid vector encoding human CLDN4 [56]. CPE was purchased from Sigma (Sigma, St. Louis, MO, USA).

### 4.4. Immunohistochemistry

Consecutive sections of 4 μm of OSCC were immunohistochemically stained using 0.2 µg/mL of primary antibodies by a previously described immunoperoxidase technique [57]. Primary antibodies were 4D3, Ki-67, p53 (DAKO, Glastrup, Denmark), E-cadherin (Transduction Laboratories, Lexington, KY), YAP, and TAZ (Abcam, Cambridge, UK). Secondary antibodies (peroxidase-conjugated anti-IgG antibodies; Medical and Biological Laboratories, Nagoya, Japan) were used at a concentration of 0.2 µg/mL. Tissue sections were color-developed with diamine benzidine hydrochloride (DAKO) and counterstained with Meyer’s hematoxylin (Sigma). As a negative control, non-immunized rat IgG (Santa Cruz Biotechnology, Santa Cruz, CA, USA) was used as the primary antibody.

### 4.5. Protein Extraction

For preparing whole cell lysate, HSC3 and HSC4 cells were washed twice with cold PBS, harvested and lysed with 0.1% SDS-added RIPA-buffer (Thermo Fisher Scientific, Tokyo, Japan) [58]. Cell fractions were extracted using a Cell Fractionation Kit (Abcam, Cambridge, MA, USA), according to the manufacturer’s instructions [59]. Protein assay was performed using a Protein Assay Rapid Kit (Wako Pure Chemical Corporation, Osaka, Japan).

### 4.6. Immunoblot Analysis

Lysates (20 μg) were subjected to immunoblot analysis using sodium dodecyl sulfate polyacrylamide gel electrophoresis (SDS-PAGE; 12.5%), followed by electrotransfer onto nitrocellulose filters. The filters were incubated with primary antibodies, followed by peroxidase-conjugated IgG antibodies (Medical and Biological Laboratories). Anti-tubulin antibody was used to assess the levels of protein loaded per lane (Oncogene Research Products, Cambridge, MA, USA). The immune complex was visualized using an Enhanced Chemiluminescence Western-blot detection system (Amersham, Aylesbury, UK). Antibodies for CLDN4 (4D3), E-cadherin (transduction), vimentin (DAKO), YAP1, phosphorylated YAP1 (pS127), ZO-1, ZO-2, MST, GAPDH (glyceraldehyde-3-phosphate dehydrogenase), cyclin D1 (CCND1) (Abcam), large tumor suppressor kinase 1 (LATS1), phosphorylated LATS (pThr1079), (Cell Signaling Technology, Beverly, MA, USA), nucleostemin (NS), CD44 (Santa Cruz Biotechnology), BRAF (Assay Biotechnology Company, Inc., Fremont, CA, USA), Snail (Biorbyt, Cambridge, UK), TAZ, BRAF^V600E^ (Abnova Corp., Taipei City, Taiwan), phosphorylated TAZ (pS89, Covalab, Cambridge, UK), β-actin (Zymed Laboratories Inc., South San Francisco, CA, USA), TEA domain family member 1 (TEAD), tubulin, and lamin (Proteintech Group Inc., Rosemont, IL, USA) were used as primary antibodies.

### 4.7. Bacterial DNA Amplification

Bacterial DNA was extracted from the OSCC specimens (10 thin-sliced paraffin-embedded tumor specimen, deparaffinized, and hydrated) using the QIAamp DNA mini kit (Qiagen, GmbH, Hilden, Germany) according to the manufacturer’s instructions. The extracted DNA samples were stored at −20 °C. PCR was carried out for 35 cycles and each cycle consisted of the following steps: denaturation (94 °C for 1 min), annealing (50 °C for 1 min) and primer extension (72 °C for 1.5 min) after initial denaturation (94 °C for 5 min). Amplified PCR products were analyzed by 1.5% agarose gel electrophoresis in Tris–Borate-EDTA buffer. The gel was stained with 0.5 μg/mL ethidium bromide. The primer sets used for the amplification of CPE DNA were as follows: forward, 5′-TCC CCT TTC TAG ATA ACG ATT AAC AC-3′ and reverse, 5′-GTT AGC ATG CTG TTT TCT AAG TTA AAA CC-3′ [60]. Primers were synthesized by Sigma Genosys (Ishikari, Japan).

### 4.8. Immunoprecipitation

Immunoprecipitation was performed according to the method described previously [61]. Briefly, whole cell lysates were pre-cleaned in lysis buffer with protein A/G agarose (Santa Cruz) for 1 h at 4 °C and subsequently centrifuged. The supernatants were incubated with antibodies against YAP1 (Abcam) or CLDN4 (4D3) and protein A/G agarose for 3 h at 4 °C. Precipitates were collected via centrifugation, washed five times with lysis buffer, solubilized with sample buffer (Sigma, 40 µg), and subjected to an immunoblot analysis.

### 4.9. Cell Surface Labeling

Cell surface proteins in IEC6 cells were iodized with Na^131^I (Amersham) with iodination reagent (Pierce, Rockford, IL, USA), which was added into the culture media and incubated with cells for 1 h. Then, cells were washed thrice with cold PBS and subjected to protein extraction as mentioned above. Radioactivity of the extracted proteins was determined using a liquid scintillation counter.

### 4.10. Enzyme-Linked Immunosorbent Assay (ELISA) for Rat CLDN4

An ELISA system was purchased from MyBiosource, Inc. (San Diego, CA, USA), and the assay was performed according to the manufacturer’s instructions.

### 4.11. Statistical Analysis

Statistical significance was calculated using chi-square, Fisher’s square test, and Kruskal-Wallis test with InStat software (GraphPad Inc., Los Angeles, CA, USA). Statistical significance was defined as a two-sided *p*-value of <0.05.

## Figures and Tables

**Table 1:** EMT phenotype and YAP1 activation in SSA/P-D.

**Tissue**	***n***	**E-Cadherin**		***P***	**CLDN4**	**Cytosol**	***P***
		**Retained**	**Decrease**		**Membrane**		
Normal	40	40	0		40	0	
TA	28	28	0		28	0	
SSA/P	25	25	0		25	0	
SSA/P-D	12	2	10	<0.0001	3	9	<0.0001
**Tissue**	***n***	**YAP1**	**Nuclear**	***P***	**TAZ**	**Nuclear**	***P***
		**Cytosol**			**Cytosol**		
Normal	40	40	0		40	0	
TA	28	28	0		28	0	
SSA/P	25	25	0		25	0	
SSA/P-D	12	3	9	<0.0001	12	0	NS

(1) E-cadherin levels were compared with that in normal mucosa. When the level was less than half of that in normal mucosa, it was considered as a decrease. (2) Subcellular localization of the protein was determined by immune histochemistry. (3) *P*-value was calculated by chi-square test. Normal, normal mucosa; TA, tubular adenoma; SSA/P, serrated sessile adenoma/polyp; SSA/P-D, SSA/P with dysplasia.

**Table 2:** *Clostridium perfringens* enterotoxin gene in SSA/P-D.

Tissue	*n*	CPE	*P*
		Positive	
Normal	40	0	
TA	28	0	
SSA/P	25	1	
SSA/P-D	12	11	<0.0001

(1) CPE gene was amplified by PCR. (2) *P*-value was calculated by chi-square test. CPE, *Clostridium perfringens* enterotoxin; Normal, normal mucosa; TA, tubular adenoma; SSA/P, serrated sessile adenoma/polyp; SSA/P-D, SSA/P with dysplasia.

**Table 3:** YAP activation and *Clostridium perfringens* enterotoxin in human CRC cases.

Case	pT/pN	BRAF^V600E^	CPE	Cytoplasmic CLDN4	Nuclear YAP
1	pT3/pN0	−	−	−	−
2	pT3/pN0	−	−	−	−
3	pT3/pN0	−	−	−	−
4	pT3/pN1	−	−	−	−
5	pT3/pN0	+	−	−	−
6	pT3/pN1	+	−	−	−
7	pT3/pN1	+	−	−	−
8	pT3/pN0	+	+	+	+
9	pT3/pN1	+	+	+	+
10	pT3/pN2	+	+	+	+

CPE, *Clostridium perfringens* enterotoxin; pT3, invasion into subserosal layer; pN1, metastasis to 1–3 regional lymph node(s); pN2, metastasis to 3 or more regional lymph nodes.

## Abbreviations

SSA/P	Sessile serrated adenoma/polyp
SSA/P-D	Sessile serrated adenoma/polyp with dysplasia
TA	uTbular adenoma
TAZ	Transcriptional coactivator with. PDZ-binding motif
ZO	Zonula occludens
MST	Mammalian Ste20-like kinases
LATS	Large tumor suppressor
TEAD	TEA domain family member
EMT	Epithelial-mesenchymal-transition
**C**-CPE	**C**-terminus domain of CPE
CLDN	claudin
YAP	yes-associated protein
